# An easy-to-use nomogram predicting overall survival of adult acute lymphoblastic leukemia

**DOI:** 10.3389/fonc.2022.977119

**Published:** 2022-09-26

**Authors:** Yu Liu, Ruyue Zheng, Yajun Liu, Lu Yang, Tao Li, Yafei Li, Zhongxing Jiang, Yanfang Liu, Chong Wang, Shujuan Wang

**Affiliations:** ^1^ Department of Hematology, The First Affiliated Hospital of Zhengzhou University, Zhengzhou, China; ^2^ Department of Orthopaedics, Rhode Island Hospital, Warren Alpert Medical School, Brown University, Providence, RI, United States

**Keywords:** acute lymphoblastic leukemia, adult, overall survival, nomogram, prognosis

## Abstract

Adult acute lymphoblastic leukemia (ALL) is heterogeneous both biologically and clinically. The outcomes of ALL have been improved with the application of children-like regimens and novel agents including immune therapy in young adults. The refractory to therapy and relapse of ALL have occurred in most adult cases. Factors affecting the prognosis of ALL include age and white blood cell (WBC) count at diagnosis. The clinical implications of genetic biomarkers, including chromosome translocation and gene mutation, have been explored in ALL. The interactions of these factors on the prediction of prognosis have not been evaluated in adult ALL. A prognostic model based on clinical and genetic abnormalities is necessary for clinical practice in the management of adult ALL. The newly diagnosed adult ALL patients were divided into the training and the validation cohort at 7:3 ratio. Factors associated with overall survival (OS) were assessed by univariate/multivariate Cox regression analyses and a signature score was assigned to each independent factor. A nomogram based on the signature score was developed and validated. The receiver operating characteristic (ROC) curve, calibration curve, and decision curve analysis (DCA) were used to assess the performance of the nomogram model. This study included a total of 229 newly diagnosed ALL patients. Five independent variables including age, WBC, bone marrow (BM) blasts, MLL rearrangement, and ICT gene mutations (carried any positive mutation of IKZF1, CREBBP and TP53) were identified as independent adverse factors for OS evaluated by the univariate, Kaplan-Meier survival and multivariate Cox regression analyses. A prognostic nomogram was built based on these factors. The areas under the ROC curve and calibration curve showed good accuracy between the predicted and observed values. The DCA curve showed that the performance of our model was superior to current risk factors. A nomogram was developed and validated based on the clinical and laboratory factors in newly diagnosed ALL patients. This model is effective to predict the overall survival of adult ALL. It is a simple and easy-to-use model that could efficiently predict the prognosis of adult ALL and is useful for decision making of treatment.

## Introduction

Acute lymphoblastic leukemia (ALL), the second most common acute leukemia in adults, originates from lymphocyte progenitor cells of B- or T-cell origin (1). In adults, more than 75% of cases develop from precursors of the B-cell lineage, with the remainder consisting of malignant T-cell precursors (2). Children aged 1-4 years and elders aged 50 years are the two peaks of age-specific incidence of ALL in a pattern of bimodal distribution (2–4). The cure rates are more than 90% in children (5). The 10-year OS rate were 86% in adolescent and young adult cases with ALL (6). The adult ALL had suboptimal outcomes with cure rates below 40% in those over the age of 40 years old (7). The five-year overall survival in patients older than 50 years old were only 25%. Cases over the age of 60 years old have extremely inferior outcomes (8). The differences in clinical responses to treatments between pediatric and adult ALL cases may be due to the poor tolerability to intensive therapy, severe chemotherapy-associated side effects and higher-risk disease features in adults (9). A prediction model taking these factors into account could be helpful to improve the overall survival rate in adult ALL.

In addition to the ages at diagnosis, impacts of clinical features on the prognosis of ALL cases have been explored. White blood cell count (WBC) and bone marrow (BM) blasts percentage have been identified as independent prognostic factors for patient’s outcomes. These hematological parameters may be associated with genetic abnormalities in ALL (10). Various chromosomal alterations in ALL are important in driving chromosomal gains and losses that may be associated with the target therapy, survival and relapse of the disease (5, 11, 12). Chromosomal abnormalities such as t (4;11) (q21;q23) are associated with poor prognosis (13, 14), whereas patients with t(12;21)(p13;q22) translocation have a better survival rate (15, 16). A subtype of ALL carrying t(9;22)(q34;q11.2) translocation had a poor prognosis, which has been improved significantly by the combination of tyrosine kinase and chemotherapy strategies (17–19). Somatic mutations of genes are important in the leukemogenesis and prognosis. Accurate identification of key genetic abnormalities is critical to disease stratification, selection of targeted agents and clinical decision of health care providers and patients. In the last few years, the application of next generation sequencing (NGS) in the detection of genomic aberrations has added new insights into the pathogenesis of ALL based on fusion genes or the altered expression of key genes (20). The type and frequency of gene mutations in ALL are not identical with AML. The clinical significance of the mutated genes is still controversial. However, patients with mutated *IKZF1* and *PAX5* had poor survivals (11).

The risk stratification of ALL was established based on clinical factors such as age, WBC, cytogenetics and the response to chemotherapy. With the clinical application of NGS approach, various disease-related and patient-specific factors including cytogenetic and molecular abnormalities, presence of central nervous system (CNS) disease, and treatment response have been used to define risk and assess prognosis for adult ALL (21). The risk stratification is important to the optimal management of ALL. However, even if patients were categorized into the same risk stratification and received the same treatment, they could still experience different treatment responses. The disparities of response to treatment suggested that the current risk stratification system of ALL might not meet the needs of clinical practice. Therefore, a predictive model for the survival of ALL based on clinical and biological characteristics is highly anticipated before treatment initiation.

Nomogram predictive model has been used in multiple malignancies including acute myeloid leukemia (22), chronic lymphocytic leukemia (23) and pediatric acute lymphoblastic leukemia (24) for progression and overall survivals. It exhibited the highest accuracy and discrimination in survival prediction comparing to the individual prognostic factors. A nomogram for the overall survival of adult ALL could be important for treatment selection and outcome prediction. This study developed a nomogram based on patient- and disease-specific parameters to predict the overall survival of adult ALL at 1-, 2-, and 3-year.

## Materials and methods

### Patients

**Figure 1:**
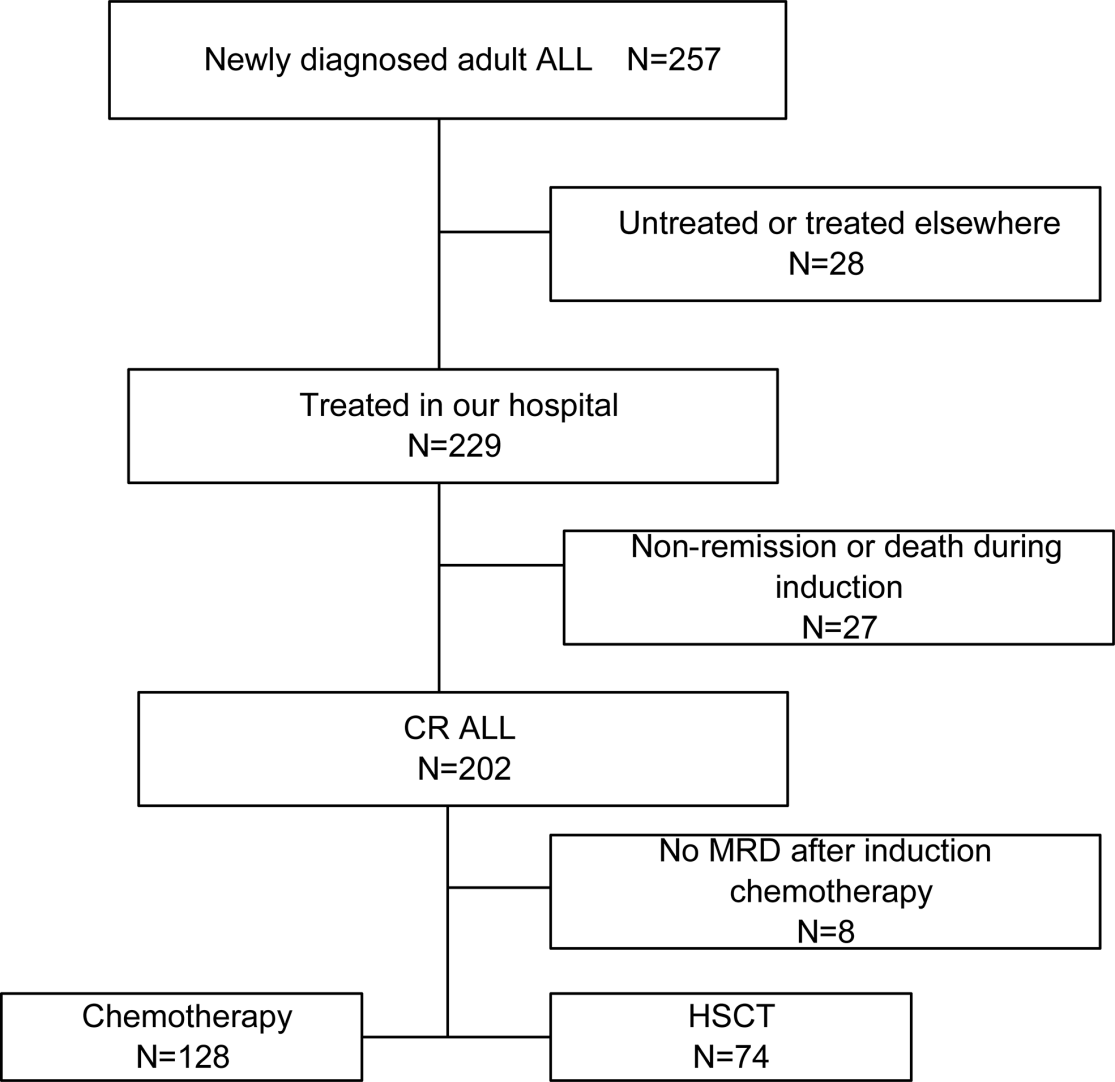
An overview flowchart containing ALL patients’ recruitment and exclusion. ALL, acute lymphoblastic leukemia; CR, complete remission; HSCT, hematopoietic stem cell transplantation.

This retrospective study included 229 ALL patients (age ≥ 14 years) that are newly diagnosed and treated at the First Affiliated Hospital of Zhengzhou University between June 2016 and May 2020. An overview flowchart containing patients’ recruitment and exclusion was shown in **Figure 1**. The cases were follow-up until death, loss of follow-up, or at November 2021. The diagnosis of ALL, complete remission (CR), relapse, and risk stratification was defined according to Chinese guidelines for diagnosis and treatment of adult acute lymphoblastic leukemia (2021) (25). Clinical information was extracted from patient medical records and mainly included gender, age, WBC, hemoglobin (HGB), platelet (PLT), peripheral blasts (PB), bone marrow (BM) blasts at diagnosis, immunophenotype, fusion gene, gene mutations and chromosomal karyotype, risk stratification, treatment regimens, complications, transplant, and survival status. These patients received the induction chemotherapy scheme including VD(CL)P regimen (vincristine, daunorubicin, cyclophosphamide, L-asparaginase or asparaginase, prednisone) or Hyper-CVAD regimen (cyclophosphamide, vincristine, Adriamycin, Dexamethasone/methotrexate, cytarabine). After achieving CR, patients received 2-4 cycles of consolidation chemotherapy (high-dose cytarabine/methotrexate/L-asparaginase or asparaginase). They then continued to receive chemotherapy until they completed six additional cycles or received an allotransplant. Maintenance therapy included 6-mercaptopurine and methotrexate. Sixty-nine patients with *BCR-ABL* fusions accepted imatinib (n=7; 600mg/d) or dasatinib (n=62; 100mg/d) in combination with chemotherapy, of which 22 (31.9%) underwent allo-HSCT. All patients received central nervous system (CNS) prophylaxis with intrathecal methotrexate, cytarabine and dexamethasone. Patients with suitable donors received allogeneic hematopoietic stem cell transplantation (HSCT) when indicated. Seventy-four subjects (32.3%) received an allotransplant, 31 (41.9%) from an HLA-identical sibling, 28 (37.8%) from an HLA-haplotype-mismatched related donor, and 15 (20.3%) from an HLA-matched unrelated donor. Busulfan and cyclophosphamide (modified Bu/CY) were used for conditioning. When the donor was not an HLA-identical sibling, 10mg/kg of rabbit anti-thymocyte globulin (r-ATG) was administered before the transplant. Donors were given recombinant human G-CSF before having their bone marrow and blood cells harvested and injected into the recipient. Cyclosporine, mycophenolate mofetil, and short-term methotrexate were used to prevent graft-vs.-host disease.

The study was approved by the Ethics Committee of the First Affiliated Hospital of Zhengzhou University in accordance with the Declaration of Helsinki. Before enrollment in this study, written informed consent was obtained from patients or legal guardians.

### Next-generation sequencing

Pre-treatment bone marrow mononuclear cells were harvested *via* density gradient centrifugation. DNA was extracted using a blood genome extraction kit (Tiangen company, Beijing, China). ALL second-generation sequencing gene chips (Yuanqi Biomedical Technology company, Shanghai, China) and MISEQ second-generation sequencer (Illumina Inc., San Diego, CA, USA) were used to detect sixteen mutation genes, including *NOTCH1*, *FBXW7*, *IKZF1*, *TP53*, *PAX5*, *FLT3*, *IL7R*, *CREBBP*, *JAK1*, *JAK2*, *JAK3*, *CRLF2*, *PHF6*, *PTEN*, *NT5C2*, and *SH2B3*.

### Fusion gene qualitative screening test

Ficoll density gradient centrifugation was used to separate pre-treatment bone marrow mononuclear cells. Total RNA was extracted using TRIzol Reagent, and the cDNA was synthesized using Reverse Transcription Kit (Shanghai Yuanqi Biomedical Technology Co.). Forty-three common fusion genes (**Table S1**) were then assessed by quantitative real-time polymerase chain reaction (qRT-PCR).

### Model and nomograms construction

First, we randomly divided the patients into a training (n = 161) and a validation (n = 68) cohort by a ratio of 7:3. The patient characteristics in the training and validation cohorts were then compared to confirm that the validation and training groups were comparable. We identified OS-associated factors in the training cohort using the univariate *Cox* proportional hazards regression (CPH) model (26). Variables with *P* < 0.05 were entered into the multivariable *Cox* proportional hazards model. These variables in the final multivariable *Cox* regression model were selected using the backward and forward stepwise method based on the Akaike information criterion (27). The independent prognostic factors determined by the multivariate analysis were used to develop a nomogram for OS.

### Validation of the model and nomograms

The signature score for each patient was calculated with multivariable *Cox* regression analysis. Patients in each cohort were divided into high-risk and low-risk groups based on the signature score cutoff value. The optimal cutoff value was determined using the X-tile (28). We applied a *log-rank* test to examine the difference in OS between the high-risk and low-risk groups. Meanwhile, the time-dependent receiver operating characteristic curve (ROC) (29) at one-year, two-year, and three-year was applied to assess the prognostication performance of the signature in the training cohort and validation cohort using the R package “timeROC” (30) and “survival” (https://CRAN.Rproject.org/package=survival). The area under curve (AUC) value of more than 0.5 shows a non-random effect, and 1 suggests a perfect model (31). The nomogram was validated with 1000 bootstrapping internally and externally, and calibrated at 1-year,2-year, and 3-year using the R package “rms” (https://CRAN.R-project.org/package=rms). Decision curve analysis (DCA) was conducted to assess the clinical application prospects of the model in the training cohort (32). Points in the nomograms are assigned based on the hierarchy of effects on survival. Calibration reveals the capacity of a model to make accurate predictions of the outcome. The observed rates *vs.* the nomogram-predicted probabilities of the models were used to generate calibration curves. Predictions in a well model were confirmed to fall on a 45° diagonal line.

### Statistical analysis

Data analysis was performed with R (version 4.1.1, Auckland, NZ, United States, http://www.r-project.org/). OS was defined as the time from diagnosis to death or loss of follow-up. The *Mann-Whitney U* test was used to compare differences in continuous variables, while the *chi-square* test or *Fisher’s* exact test was used to identify the differences in categorical variables. OS was estimated using the *Kaplan-Meier* survival analysis, and the differences in survival curves were compared using the *log-rank* test. *Cox* proportional hazards models were used to assess the clinical factors with survival. Meanwhile, the hazard ratio (*HR*) and corresponding 95% confidence interval (*CI*) were calculated. Two-sided *P* values less than 0.05 were considered statistically significant.

## Results

### Clinical information of ALL patients

There were 257 ALL cases who were admitted and screened in our hospital, and 28 cases were excluded due to their refusal to treatment. Among the 229 cases treated in our hospital, 27 failed to achieve remission or died during induction, and 202 cases achieved complete remission. One hundred twenty-eight received chemotherapies only, while 74 received an allo-HSCT as consolidation. We eventually included the 229 cases treated in our hospital in the prediction model analysis. The median age of the cases was 50 (Inter Quartile Range, IQR, 17-44) years old; with 42.4% (97/229) of male and 81.2% (186/229) of B-cell ALL. There were 125 (54.6%) cases categorized into high risk. The median WBC was 18 (IQR, 4.9-73.5) ×10^9^/L. The median PLT was 45 (IQR, 18-87) ×10^9^/L. The median BM blasts were 90 (IQR, 81.2-94) %. The median peripheral blasts (PB) of the patients was 57 (IQR, 8-83)% at diagnosis. Moreover, 99 (43.2) % were dead. The median follow-up duration was 773 (IQR, 741.8-804.2) days. The median OS was 811 (95% *CI*: unavailable) days. The 2-year OS rate was 50.6% (95% *CI*: 43.4% -57.9%). Furthermore, 37.6% (70/186) patients underwent HSCT in first CR.

### Mutational landscape of adult ALL patients

**Figure 2:**
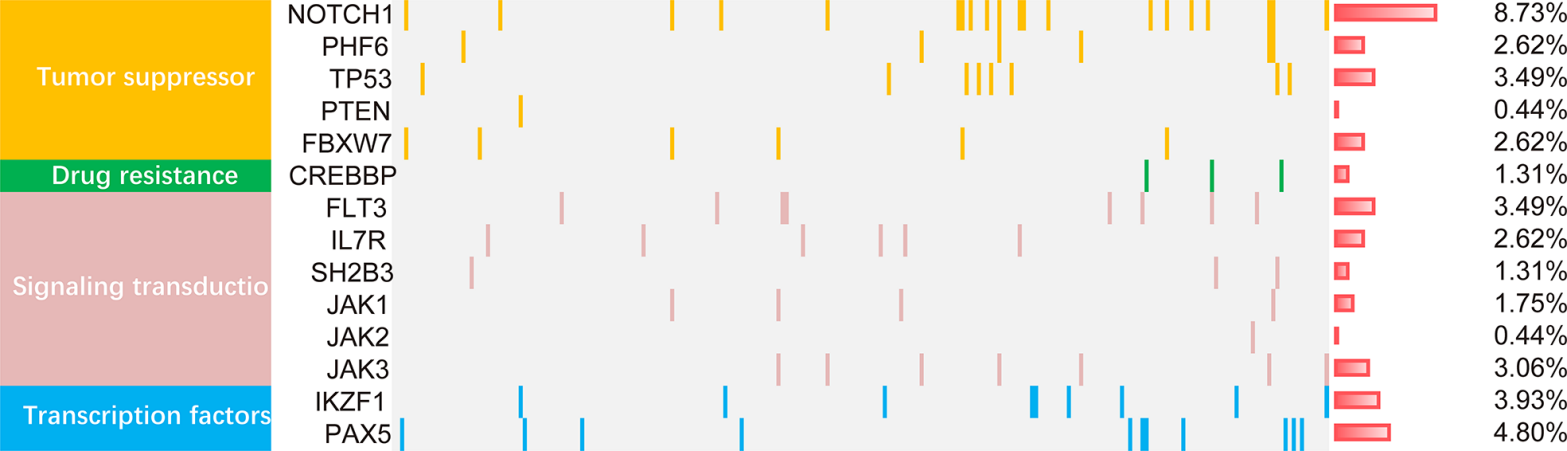
The mutational landscape of 229 ALL patients. The landscape displayed all genetic anomalies for each subject. A single patient instance was represented by the boxes in one column. Mutations were color coded according to mutation type. The frequency distribution of all aberrations was depicted by the histogram on the right.

Mutations were detected in 14 genes from the 229 patients (**Figure 2**). The frequent mutations were found in *NOTCH1* (8.73%), *PAX5* (4.80%), *IKZF1* (3.93%), *TP53* (3.49%), *FLT3* (3.49%) and *JAK3* (3.06%). Furthermore, the genes were categorized by function into tumor suppressor genes (17.9%), transduction genes (12.66%), signaling transcription factors gene (8.73%), and drug resistance genes (1.3%). Univariate analysis of the mutated genes was performed in all cases. The results showed that mutations in *IKZF1*, *CREBBP* and *TP53* genes had prognostic significance. Since the low incidence of mutations, any positive of the above three gene mutations was defined as *ICT* gene mutations positive. These gene mutations were applied in the development of the nomogram model.

### Prognostic *Cox* model and nomogram construction

The training cohort included 161 ALL cases with 66 females and 95 males. The validation cohort included 68 ALL cases with 31 females and 37 males. Clinical information was categorized according to the cutoff value based on X-tile (age, 48 years; WBC, 30×10^9^/L; PLT, 30×10^9^/L; HGB, 100 g/L; BM blast, 90%; PB, 70%). There were no statistically significant variations between the training and validation cohorts regarding clinical characteristics (**Table 1**).

**Table 1:** Comparision of the training cohort and the validation cohort.

Variable	ALL	Training	Validation			*P*-value
	NO.	161	68			
Male	97 (42%)	66 (40.99%)	31 (45.59%)			0.62
Age≥48 years	44 (19%)	28 (17.39%)	16 (23.53%)			0.37
B-ALL	186 (81%)	135 (83.85%)	51 (75%)			0.17
WBC≥30×10^9^/L	95 (41%)	66 (40.99%)	29 (42.65%)			0.93
HGB≥100g/L	64 (28%)	49 (30.43%)	15 (22.06%)			0.26
PLT≥30×10^9^/L	144 (63%)	105 (65.22%)	39 (57.35%)			0.33
PB≥70%	102 (45%)	69 (42.86%)	33 (48.53%)			0.52
*BCR-ABL+*	69 (30%)	47 (29.19%)	22 (32.35%)			0.75
*MLL+*	12 (5%)	8 (4.97%)	4 (5.88%)			1
*E2A-PBX*+	5 (2%)	5 (3.11%)	0 (0%)			0.33
*SET-CAN*+	3 (1%)	2 (1.24%)	1 (1.47%)			1
*SIL-TAL1*+	4 (2%)	2 (1.24%)	2 (2.94%)			0.73
BM-blast	118 (52%)	77 (47.83%)	41 (60.29%)			0.11
Risk	125 (55%)	82 (50.93%)	43 (63.24%)			0.12
CR	202 (88%)	146 (90.68%)	56 (82.35%)			0.12
HSCT	74 (32%)	54 (33.54%)	20 (29.41%)			0.65
*ICT* gene	20 (9%)	13 (8.07%)	7 (10.29%)			0.77
*NOTCH1*	20 (9%)	14 (8.7%)	6 (8.82%)			1
*FBXW7*	6 (3%)	4 (2.48%)	2 (2.94%)			1
*IL7R*	6 (3%)	6 (3.73%)	0 (0%)			0.25
*IKZF1*	9 (4%)	7 (4.35%)	2 (2.94%)			0.9
*FLT3*	8 (3%)	5 (3.11%)	3 (4.41%)			0.92
*TP53*	8 (3%)	4 (2.48%)	4 (5.88%)			0.38
*PAX5*	11 (5%)	9 (5.59%)	2 (2.94%)			0.6
*SH2B3*	3 (1%)	2 (1.24%)	1 (1.47%)			1
*JAK2*	1 (0%)	1 (0.62%)	0 (0%)			1
*PHF6*	6 (3%)	3 (1.86%)	3 (4.41%)			0.52
*JAK3*	7 (3%)	5 (3.11%)	2 (2.94%)			1
*JAK1*	4 (2%)	2 (1.24%)	2 (2.94%)			0.73
*PTEN*	1 (0%)	1 (0.62%)	0 (0%)			1
*CREBBP*	3 (1%)	2 (1.24%)	1 (1.47%)			1
Dead	99 (43%)	70 (43.48%)	29 (42.65%)			1
OS, median (interquartile range, days)	438 (184 to 773)	525.96 (384.06)		494.18 (429.68)		0.29

WBC, white blood cell counts; HGB, hemoglobin; PLT, platelet; BM, bone marrow; PB, peripheral blood; CR, complete remission; ICT gene, if carried one or more of IKZF1, CREBBP and TP53; T.B,T acute lymphoblastic leukemia or B acute lymphoblastic leukemia; HSCT, hematopoietic stem cell transplantation.

**Figure 3:**
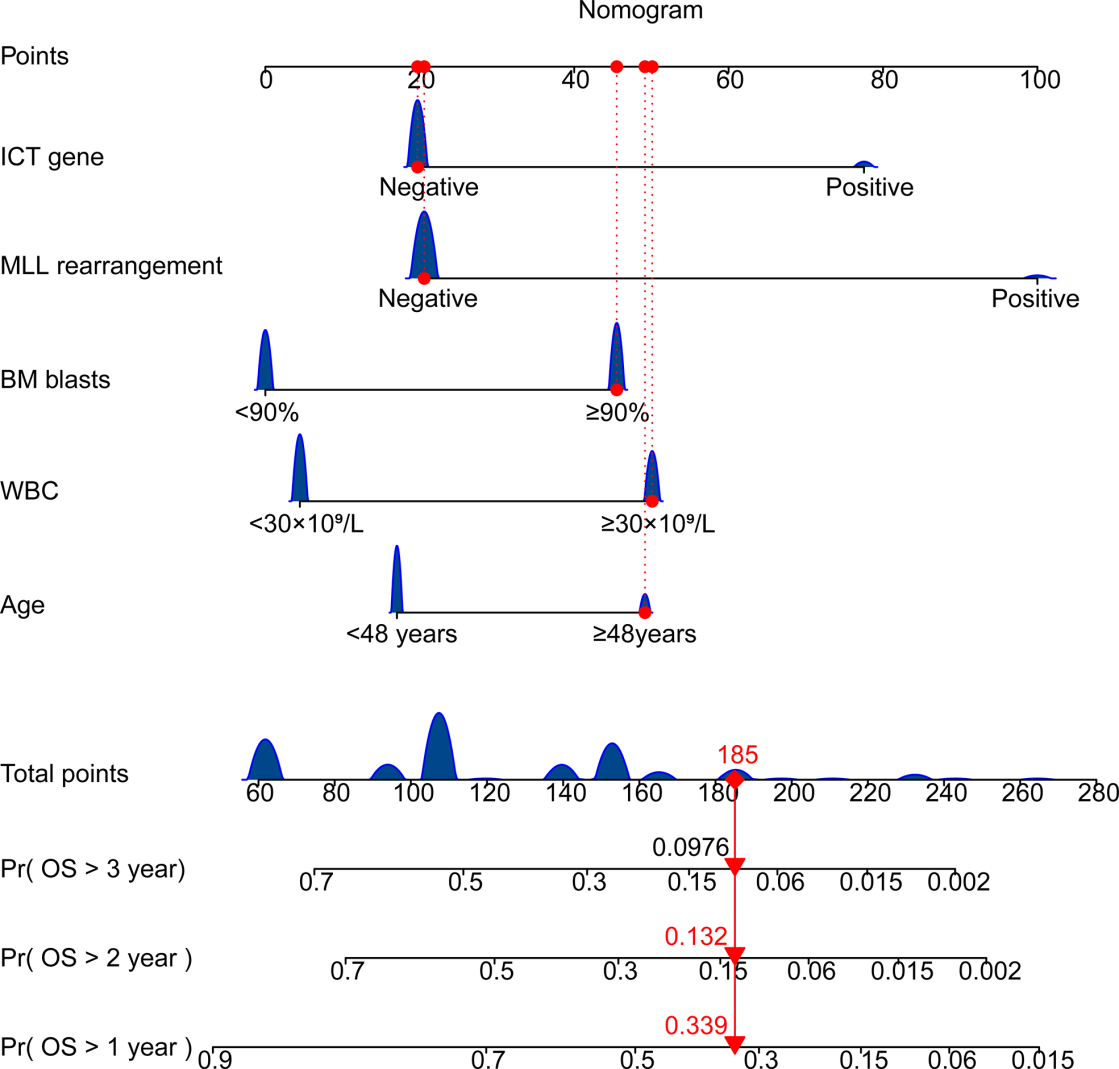
Nomogram for predicting overall survival of patients with ALL. Age ≥ 48 years; WBC ≥ 30 × 10^9^/L; BM blast ≥ 90%; the presence of *MLL* rearrangement; ICT gene if carried one or more of *IKZF1*, *CREBBP* and *TP53*. BM, bone marrow; WBC, white blood cell counts; OS, overall survival.

Univariate *Cox* regression was used to identify factors associated with ALL prognoses (**Table 2**). There were six factors with prognostic significance. Stepwise regression analysis was applied to evaluate the factors and confirmed that the combination of five factors could be used to predict prognosis. These factors are age, WBC, *MLL* rearrangement, BM blast, and ICT gene. A risk score system was developed based on these factors. In addition, we combined these factors to develop a nomogram that predicted ALL patients’ 1-year survival probability, 2-year survival probability, and 3-year survival probability. Each variable corresponds to a score on the Points line in the nomogram. The sum of the scores relating to all variables also has a score on the “Total points” line. Then the 1-year survival probability, 2-year survival probability, and 3-year survival probability of a patient can be estimated by the score on the “Total points” line (**Figure 3**). A risk scoring model was developed to incorporate the weighted coefficients of the variables: 1.726 × Age + 2.171 × WBC + 2.167 × BM blast + 3.855 × *MLL* + 2.671× ICT gene (Age ≥ 48 years; WBC ≥ 30 × 10^9^/L; BM blast ≥ 90%; the presence of *MLL* rearrangement; ICT gene if carried one or more of *IKZF1*, *CREBBP* and *TP53*). A prognostic nomogram that integrated all five significantly independent factors from the *Cox* regression model was developed (**Figure 3**).

**Table 2:** Univariate and multivariate analysis.

Characteristics	Univariate analysis		Multivariable analysis	
	*HR* (95%*CI*)	*P* Value	*HR* (95%*CI*)	*P* Value
**Age**	**1.75 (1.01-3.01)**	**0.045**	**1.73 (1-2.99)**	**0.052**
Sex	1.05 (0.64-1.71)	0.853	NA	NA
**WBC**	**2.06 (1.27-3.34)**	**0.004**	**2.17 (1.32-3.56)**	**0.002**
HGB	0.63 (0.36-1.1)	0.104	NA	NA
PLT	0.94 (0.57-1.54)	0.802	NA	NA
PB	1.65 (1.01-2.68)	0.046	NA	NA
**BM_blasts**	**2.21 (1.33-3.68)**	**0.002**	**2.17 (1.29-3.63)**	**0.003**
T.B	1.21 (0.65-2.27)	0.543	NA	NA
*BCR-ABL*	0.73 (0.42-1.25)	0.253	NA	NA
***MLL***	**3.21 (1.16-8.9)**	**0.025**	**3.85 (1.34-11.12)**	**0.013**
*E2A-PBX*	0.58 (0.08-4.16)	0.585	NA	NA
*SET-CAN*	0.63 (0.09-4.52)	0.643	NA	NA
*SIL-TAL1*	0.73 (0.1-5.24)	0.751	NA	NA
**ICT gene**	**2.53 (1.13-5.67)**	**0.024**	**2.67 (1.17-6.09)**	**0.02**
*IKZF1*	2.85 (0.88-9.26)	0.082	NA	NA
*CREBBP*	0 (0-Inf)	0.996	NA	NA
*TP53*	2.55 (0.91-7.09)	0.074	NA	NA
*FBXW7*	0.95 (0.13-6.85)	0.958	NA	NA
*FLT3*	2.39 (0.75-7.65)	0.141	NA	NA
*IL7R*	0 (0-Inf)	0.996	NA	NA
*JAK1*	0 (0-Inf)	0.995	NA	NA
*JAK2*	0 (0-Inf)	0.996	NA	NA
*JAK3*	0.93 (0.23-3.79)	0.916	NA	NA
*NOTCH1*	1.46 (0.53-4.04)	0.464	NA	NA
*PAX5*	0.36 (0.05-2.58)	0.308	NA	NA
*PHF6*	0.38 (0.05-2.75)	0.339	NA	NA
*PTEN*	NA	NA	NA	NA
*SH2B3*	0.66 (0.09-4.77)	0.681	NA	NA
Risk	0.95 (0.59-1.54)	0.842	NA	NA

WBC, white blood cell counts; HGB, hemoglobin; PLT, platelet; BM, bone marrow; PB, peripheral blood; CR, complete remission; ICT gene, if carried one or more of IKZF1, CREBBP and TP53; T.B,T acute lymphoblastic leukemia or B acute lymphoblastic leukemia.Bold numbers are the factors we included in the prognostic model.

### Prediction value of the model

We then investigated the performance of the model, primarily focusing on two indicators: The *P*-value of the *KM* analysis (*log-rank* test) for assessing a model’s ability to discriminate between cases with high and low risk, and the AUC value for estimating the accuracy of the model.

**Figure 4:**
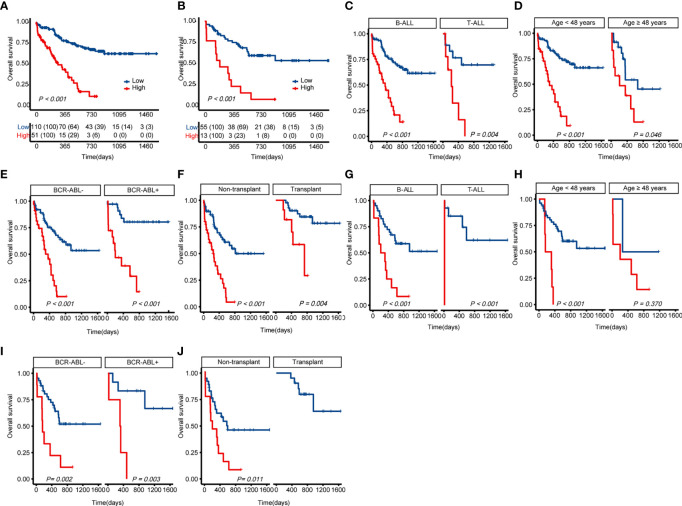
Kaplan-Meier curves for overall survival based on the predicted risk score of ALL patients. **(A)** OS of the training cohort. **(B)** OS of the validation cohort. **(C)** Differences in OS between T-ALL and B-ALL groups in the training cohort. **(D)** Differences in OS between age subgroup in the training cohort. **(E)** Differences in OS between ph+/- subgroup in the training cohort. **(F)** Differences in OS between the transplant and non-transplant groups in the training cohort. **(G)** Differences in OS between T-ALL and B-ALL groups in the validation cohort. **(H)** Differences in OS between age subgroup in the validation cohort. **(I)** Differences in OS between ph+/- subgroup in the validation cohort. **(J)** Differences in OS between the transplant and non-transplant groups in the validation cohort. The *P*-value for *Kaplan-Meier* curves is calculated by the *log-rank* test. OS, overall survival.

Cases with ALL in the training cohort were categorized into two subgroups based on the risk score (X-tile): low-risk (L-R, score < 3.91, n = 110) and high risk (H-R, score ≥3.91, n = 51) groups. As shown in **Figure 4A**, in the training cohort, the 1-year OS for the L-R and H-R groups was 78% (95%*CI* 69.8%-86.2%) and 43.4% (28.1%-58.3%), respectively (*P* < 0.001). The 2-year OS for the L-R and H-R groups was 67.4% (57.6%-76.8%) and 11.4% (0-23.9%), respectively (*P* < 0.001). Similar results were obtained in the validation cohort of 68 patients (L-R, n = 55 and H-R, n = 13; 1-year OS 77.0% [65.4%-88.6%] *vs.* 23.1% [0.2%-46.0%], *P* < 0.001; 2-year OS 59.6% [45.7%-73.5%] *vs.* 7.7% [0-22.2%], *P* < 0.001; **Figure 4B**). We performed a subgroup analysis of the training and validation cohorts, including transplant, age, T-ALL/B-ALL, and *BCR-ABL* +/**-**. The results indicated that in B-ALL and T-ALL, H-R group had a worse prognosis (**Figures 4C, G** **)**. The H-R group also had a poor prognosis in different age groups (**Figures 4D, H** **)**. Regardless of whether the cases are positive for BCR-ABL, L-R group had a favorable prognosis in general (**Figures 4E, I** **)**. Both patients with or without transplantation had a favorable prognosis in the L-R group (**Figures 4F, J** **)**. Due to a small number of patients in the validation cohort, no high-risk case was identified in the transplant group (**Figure 4J**).

**Figure 5:**
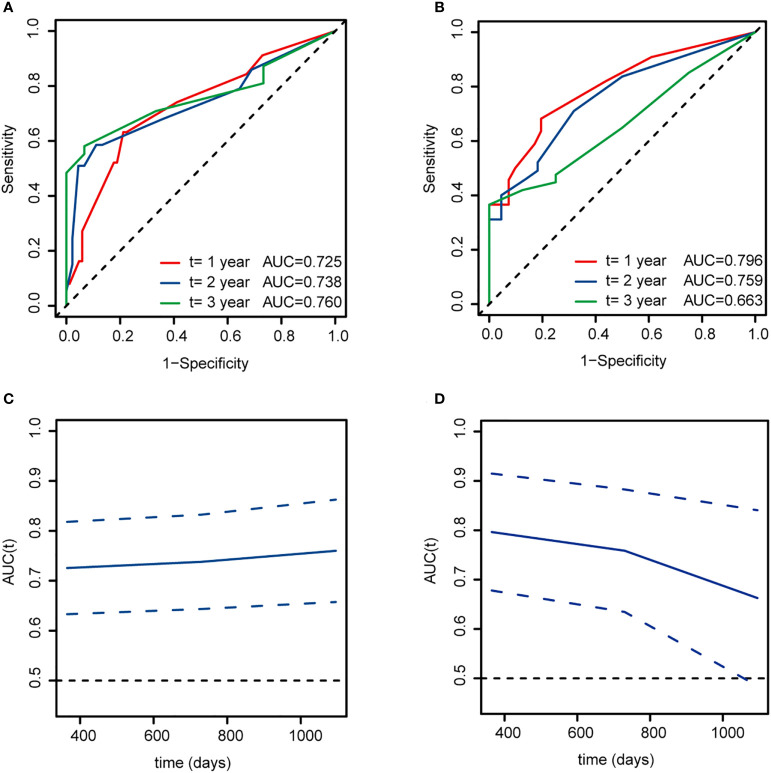
Time-dependent ROC curves for overall survival at 1, 2, and 3 years based on this model of ALL patients. **(A, C)** The training cohort. **(B, D)** The validation cohort. AUC, area under curve.

The 1-, 2-, and 3-year AUC values for this model in the training cohort were 0.725, 0.738 and 0.760, respectively (**Figure 5A**). The AUCs at 1-, 2-, and 3-year in the validation cohort were 0.796, 0.759, and 0.663, respectively (**Figure 5B**). As shown in **Figures 5C, D**, the time-dependent ROC curve suggested that the model performed well in predicting the OS of ALL cases in the training and validation cohorts.

### Calibration plot of the training and validation cohort

**Figure 6:**
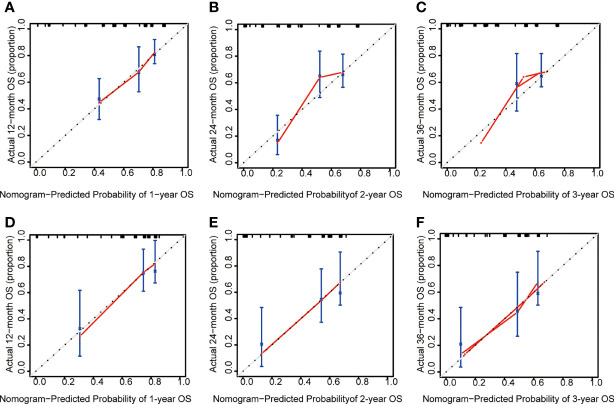
The calibration plot of ALL patients. **(A)** Calibration analysis of the training cohort at 1-year. **(B)** Calibration analysis of the training cohort at 2-year. **(C)** Calibration analysis of the training cohort at 3-year. **(D)** Calibration analysis of the validation cohort at 1-year. **(E)** Calibration analysis of the validation cohort at 2-year. **(F)** Calibration analysis of the validation cohort at 3-year. OS, overall survival.

Calibration reveals the capacity of a model to make accurate predictions of the outcome. The observed rates *vs.* the nomogram-predicted probabilities of the models were used to generate calibration curves. Predictions in a well-fitted model are verified to fall on a 45° diagonal line. The calibration plot closely resembled the ideal diagonal curve at 1-year, 2-year, and 3-year in the training (**Figures 6A–C**) and validation cohorts (**Figures 6D–F**).

### DCA plot and comparison of the model with published predictive models for prognostic assessment

**Figure 7:**
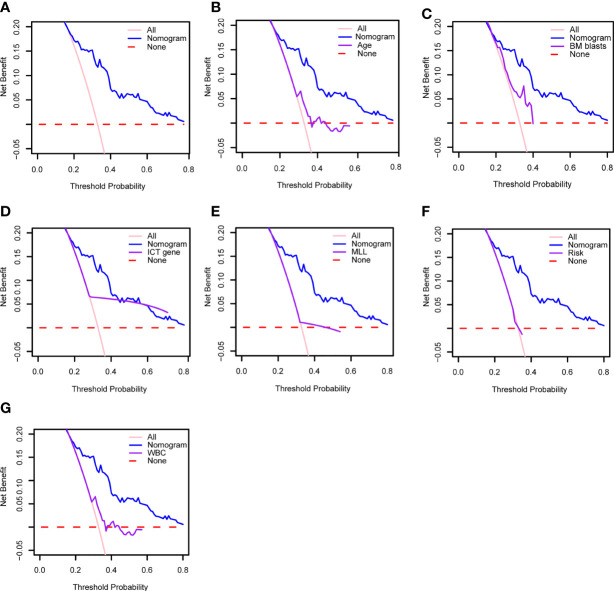
**(A)** Decision curve analysis of the clinical use of our model based on the nomogram. Decision curve analysis of the prediction value comparison between our model and these single indicators: **(B)** age, **(C)** BM blasts, **(D)** ICT gene, **(E)** MLL, **(F)** risk and **(G)** WBC. WBC, white blood cell counts; BM, bone marrow; ICT gene, if carried one or more of IKZF1, CREBBP and TP53.

Moreover, as shown in **Figure 7A**, we have performed DCA of the nomogram in all patients. The results demonstrated that the nomogram model had an excellent net benefit for 1-, 2- and 3-year OS. Meanwhile, we compared the prediction effect of the combination model with that of each as a single indicator model through DCA curves, including age, BM blast, ICT gene, *MLL* rearrangement, risk, and WBC. Our model had the highest net benefit at the threshold probabilities along the x-axis compared with other single factor models. The results showed that the performance of our model is better, as indicated in **Figures 7B–G**.

## Discussions

At present, some of the adult ALL patients end up in refractory or relapse due to the lack of accurate risk stratification, though pediatric inspired regimens are applied in young adult cases. It is particularly critical to identify patients at high-risk who are resistant to chemotherapy or have early relapse. In this study, we developed a predictive model for overall survival in the training cohort and validated it in the internal testing based on clinical information from 229 cases of adult ALL. Since there are many factors affecting ALL prognosis, we analyzed the factors associated to the overall survival by univariate analysis, and then incorporated them into multivariate analyses. We identified five risk factors through the stepwise regression method: age, WBC, *MLL* rearrangement, BM blast, and ICT gene at diagnosis. A risk score was established for each factor based on multivariate analyses. These factors were integrated into the model for better performance parameters. Subsequently, a nomogram of adult ALL, including these factors, was developed and validated to predict the OS of adult ALL patients. This nomogram is superior to individual risk factors and current risk stratification in the prediction of overall survival in adult ALL.

Adult ALL accounts for a relatively small proportion, but it has a low survival rate and a poor long-term prognosis. Those aged 55 years old and above have high death rates with the median age of death from ALL being 56 years old, and overall survival at 5 year is under 25%.  (33) Consistent with previous studies, age is a significant prognostic factor for adult ALL. In this study, the age was divided into two groups: < 48 years and≥48 years based on the cutoff optimization of the OS. The results demonstrated that patients younger than 48 years old had a better prognosis. Moreover, the overall survival in low-risk is favorable and not affected by patient’s age. According to the cutoff optimization, the WBC counts of patients in this study were divided into < 30 × 10^9^/L and ≥ 30 × 10^9^/L. This is consistent with the WBC cutoff proposed in NCCN guidance for ALL. These findings support the adverse risk significance of leukocytosis in ALL.

Chromosomal alteration and gene mutations play an essential role in ALL. Fusion genes are formed by translocation and rearrangement of chromosomes. Many ALL-related fusion genes have been detected, which play important roles in the pathogenesis, diagnosis, prognosis prediction, and treatment response (34). *BCR-ABL* fusion gene used to be an unfavorable factor in adult ALL (35) (36). However, the application of imatinib and other TKIs in the treatment of Philadelphia chromosome positive ALL has significantly improved patient outcomes. Therefore, patients with *BCR-ABL* fusion gene are no longer considered a high-risk group (37–39). In this article, *MLL* rearrangements were identified as an important prognostic factor in adult ALL. *MLL*-rearranged ALL (*MLL*-r-ALL) is related to aggressive biology with early relapse, a relative high incidence of central nervous system leukemia (CNSL) involvement, and poor prognosis (7, 40). The incidence of *MLL*-r-ALL is bimodal and increases with age in adults (14). *IKZF1* deletion is a critical poor prognostic biomarker in ALL (41), the mutation of which drives normal lymphocytes to develop into leukemia (42).It was reported that HSCT could improve clinical outcomes of patients with *IKZF1* mutation (43). While *CREBBP* mutation is associated with drug resistance and recurrence in ALL (44, 45). The mutation of this gene frequently occurred in childhood ALL and was rare in adults (46). The results in this study suggest that *CREBBP* mutation is associated with poor overall survival of adult ALL. B-ALL patients with *TP53* gene mutation have a higher recurrence rate. *TP53* mutation is a potential biomarker associated with poor prognosis in B-ALL patients (47–50). Allo-HSCT and the infusion of donor-derived CAR-T cells can overcome the adverse impact of *TP53* mutation on prognosis (51–53). In adult acute lymphocyte leukemia, the incidence of *IKZF1*, *CREBBP* and *TP53* was low, and our research showed that these genes were associated with prognosis. After dividing our cohort into training cohort and validation cohort, there were too few patients with these mutations. Therefore, we defined any gene mutation positive of these mutations as *ICT* gene mutations positive. Consistent with the above findings, our nomogram model incorporated *MLL* rearrangement and ICT gene mutations as weighted factors. However, *BCR-ABL* fusion genes were not weighted in this nomogram due to its prognostic impacts that can be overcome by addition of TKIs to treatment regimen.

The advantage of our research is that all the data were collected from clinical cases. This is a real-world study to evaluate the clinical accuracy of a prognostic model for adult ALL patients. The prognostic factors involved in our model could be collected in most cases at initial diagnosis. It means that this nomogram model is simple and easy to use. This model could predict overall survival of adult ALL patients more accurately than any single prognostic factor.

However, this study also has some limitations. Firstly, we lack external validation, leading to limitations in extrapolating the study findings. Secondly, the number of cases is not particularly large. Multi-center verification with a large sample size could provide more evidence for clinical application. Thirdly, some factors associated to prognosis are not included in this nomogram model. For example, CNSL is one of the main reasons of recurrence, which will affect the prognosis of ALL patients (25). However, based on the situation that the incidence of CNSL is relatively low at initial diagnosis of adult ALL, we could not carry out statistical analysis in this study. Philadelphia chromosome (Ph)-like gene testing had not be available at our hospital until last year, so early cases lacked Ph-like gene results. Fourth, the endpoint of our prediction model is OS, and further research is needed to explore the prediction for RFS (relapse-free survival).

In conclusion, we developed a simple and easy-to-use nomogram model that could predict the overall survival of adult ALL patients and be useful to guide treatment selection.

## Data availability statement

The raw data supporting the conclusions of this article will be made available by the authors, without undue reservation.

## Ethics statement

The studies involving human participants were reviewed and approved by the Ethics Committee of the First Affiliated Hospital of Zhengzhou University. Written informed consent for participation was not required for this study in accordance with the national legislation and the institutional requirements.

## Author contributions

Conceptualization: YanL, CW and SW. Data curation: YuL, RZ and LY. Formal analysis: YuL and SW. Funding acquisition: SW. Methodology: YuL, TL, YaL and SW. Project administration: ZJ, YanL, CW and SW. Software: YuL and SW. Validation: YuL and SW. Writing – original draft: YuL. Writing – review & editing, YajL, YanL and SW. All authors contributed to the article and approved the submitted version.

## Funding

This work was supported by the National Natural Science Foundation of China [grant number 81800137] and Henan Medical Science and Technology Research Project (grant number 2018020068).

## Acknowledgments

We thank all the treating physicians for allowing us to enroll their patients and the patients for allowing us to analyze their data.

## Conflict of interest

The authors declare that the research was conducted in the absence of any commercial or financial relationships that could be construed as a potential conflict of interest.

## Publisher’s note

All claims expressed in this article are solely those of the authors and do not necessarily represent those of their affiliated organizations, or those of the publisher, the editors and the reviewers. Any product that may be evaluated in this article, or claim that may be made by its manufacturer, is not guaranteed or endorsed by the publisher.
