# Supplementary material for: An easy-to-use nomogram predicting overall survival of adult acute lymphoblastic leukemia
Source: Front Oncol. 2022 Sep 26;12:977119. doi: 10.3389/fonc.2022.977119 (PMC9549528; doi:10.3389/fonc.2022.977119)
Supplement: Supplementary file 1 [file Table_1.docx]

Supplementary Material

# Supplementary Tables

**Table S1.** Assessed forty-three common fusion genes

| **Forty-three common fusion genes** | | | | | | |
| --- | --- | --- | --- | --- | --- | --- |
| *BCR-ABL* |  | *STAT5b-RARa* |  | *PML-RARa* |  | *E2A-HLF* |
| *SlL-TALl* |  | *AML1-MDS1/EV11* |  | *E2A-PBXl* |  | *AML1-ETO* |
| *MLL-AF9* |  | *MLL-(AF6、AF1O、ELL、ENL)* | | *TEL-PDGFRB* |  | *NPM-MLFl* |
| *PLZF-RARa* | | *AML1-MTG16* |  | *CBFB-MYH11* |  | *TEL-JAK2* |
| *TEL-ABL* |  | *NUP98-(HOXA9、HOXA13、*  *HOXA11、 HOXC11、HOXD13、PMX1)* | | *SET-CAN* |  | *DEK-CAN* |
| *NPM-ALK* |  | *MLL-(AFl7、AFlq、AFlp、AFX、SEPT6)* | | *TLS-ERG* |  | *ETV6-PDGFRA* |
| *TEL-AMLl* |  | *(NPM、FIP1L1、PRKAR1A、NUMA1)-RARa* | | *MLL-AF4* |  | *AML1-MTG16* |
